# Repurposing of the small-molecule adrenoreceptor-inhibitor carvedilol for treatment of the fibrotic lung

**DOI:** 10.3389/fphar.2025.1534989

**Published:** 2025-05-22

**Authors:** Maria Jordan, Kevin Schmidt, Maximilian Fuchs, Annette Just, Angelika Pfanne, Lena Willmer, Lavinia Neubert, Christopher Werlein, Patrick Zardo, Andreas Pich, Thomas Thum, Jan Fiedler

**Affiliations:** ^1^ Preclinical Pharmacology and Toxicology, Fraunhofer Institute for Toxicology and Experimental Medicine ITEM, Hannover, Germany; ^2^ Fraunhofer Cluster of Excellence for Immune Mediated Diseases (CIMD), Hannover, Germany; ^3^ Institute of Molecular and Translational Therapeutic Strategies (IMTTS), Hannover Medical School, Hannover, Germany; ^4^ Institute of Pathology, Hannover Medical School, Hannover, Germany; ^5^ Department of Cardiothoracic Transplantation and Vascular Surgery, Hannover Medical School, Hannover, Germany; ^6^ Institute of Toxicology and Core Unit Proteomics, Hannover Medical School, Hannover, Germany; ^7^ Center for Translational Regenerative Medicine, Hannover Medical School, Hannover, Germany

**Keywords:** carvedilol, idiopathic pulmonary fibrosis, fibroblast, precision-cut lung slices, drug repurposing, miR-21

## Abstract

**Introduction:**

Idiopathic pulmonary fibrosis (IPF) is a chronic fibrotic lung disease with high mortality. Current therapies are very limited, with nintedanib and pirfenidone being the only non-invasive but non-curative interventions, ultimately bridging to lung transplantation.

**Methods:**

*In silico* modeling of dysregulated pathways in IPF and screening for putative interfering small molecules identified carvedilol as a promising anti-fibrotic agent. We validated drug-mediated effects on key features of fibroblast activation in functional assays and gene expression analyses in human embryonic lung fibroblasts (MRC-5). Precision-cut lung slices (PCLSs) generated from human lung tissue were assessed for secreted fibrotic markers’ expression.

**Results:**

Treatment with carvedilol reduced metabolic activity, inhibited cell proliferation, and led to decreased migratory activity, as observed in scratch wound assays, in human lung fibroblasts. The functional profile was reflected at the transcriptional level as commonly known fibrotic marker genes, e.g., alpha smooth muscle actin and collagen 1, were robustly repressed. Proteomic profiling underlined a strong extracellular matrix interference with elevated syntheses of several collagen types and various integrins, which play a critical role in pro-fibrotic downstream signaling. Comparison of healthy and fibrotic lung tissue validated an upregulation of pro-fibrotic miR-21 secretion in the *ex vivo* PCLS model, which remained unchanged upon carvedilol therapy.

**Conclusion:**

Herein, carvedilol demonstrated significant anti-fibrotic effects on human lung fibroblasts *in vitro*, thus presenting great potential as an anti-IPF treatment. In addition, miR-21 was validated as a secreted pro-fibrotic biomarker in the *ex vivo* PCLS model.

## 1 Introduction

Idiopathic pulmonary fibrosis (IPF) is a chronic, progressive, fibrotic interstitial lung disease of unknown etiological factors with poor prognosis (Lederer et al., 2018), currently affecting roughly 3.5 million people worldwide (Maher et al., 2021). Once diagnosed, the median life expectancy is 3.8 years if not treated with anti-fibrotic medications such as nintedanib or pirfenidone (Raghu et al., 2014) that counteract the characteristic scarring of lung tissue and irreversible loss of function (Raghu et al., 2011). Although the causative events for IPF pathogenesis have not been fully revealed so far, disease progression is crucially dependent on aberrant fibroblast activation. In brief, repetitive injury of alveolar epithelia consisting of alveolar epithelial cells type 1 (AT1) and type 2 (AT2) (Desai et al., 2014; McKleroy and Lyn-Kew, 2018) leads to their pro-fibrotic reprogramming and activation (Martinez et al., 2017). Thus, dysregulated AT2 secretes pro-fibrotic cytokines and growth factors, including platelet-derived growth factor and transforming growth factor β (TGFβ), ensuing in epithelial-to-mesenchymal transition (EMT), loss of AT1, and recruitment of fibroblasts to the alveolar area (Königshoff et al., 2009; Selman and Pardo, 2006). The differentiation of fibroblasts into proliferating and migrating myofibroblasts causes exaggerated extracellular matrix (ECM) deposition, initiating the formation of fibroblastic foci (Cavazza et al., 2010; Katzenstein et al., 2008; Richeldi et al., 2017).

Unfortunately, current therapeutic measures for IPF patients cannot reverse scarring of the lung tissue. Thus, the primary goal is to improve the quality of life of IPF patients and slow down fibrosis progression, while balancing potential risks and benefits (Raghu et al., 2011). Pharmacological treatment options are restricted to the Food and Drug Administration (FDA)-approved drugs nintedanib and pirfenidone, which, however, have tolerability issues and do no stabilize or improve the lung function (Kreuter et al., 2015). Nintedanib is an orally administered tyrosine kinase inhibitor that targets fibroblast growth factor receptors (FGFRs), vascular endothelial growth factor receptors (VEGFRs), and platelet-derived growth factor receptors (PDGFRs) (Hilberg et al., 2008), affecting migration and proliferation of fibroblasts, along with their differentiation into myofibroblasts (Spagnolo et al., 2021). Expectedly, due to the cytostatic activity of nintedanib, common chemotherapeutic side effects including diarrhea, nausea, and vomiting have been reported (Corte et al., 2015; Richeldi et al., 2014). In contrast, the synthetic compound pirfenidone reduces oxidative stress, inhibits synthesis and secretion of inflammatory cytokines, and targets pro-fibrotic growth factor signaling, including TGFβ (Oku et al., 2008; Ruwanpura et al., 2020). However, although treatment with nintedanib or pirfenidone significantly decelerates fibrosis progression and reduces the risk of deterioration of lung function, neither drug showed a survival benefit (Canestaro et al., 2016). In addition, the fact that only few IPF patients are suitable for lung transplantation, which can increase the 5-year survival rate post-transplantation by approximately 50% (Keating et al., 2009), implies the continuation of an unmet need for medications curing IPF (Spagnolo et al., 2021).

In recent years, multiple studies have been conducted to search for novel compounds in that regard. Maher et al. showed that inhaled αvβ6 integrin inhibitor GSK3008348 targets pro-fibrotic regions in the lungs of IPF patients (ClinicalTrials, 2024b). However, although anti-fibrotic effects were proven in various IPF models (John et al., 2020), the efficacy of GSK3008348 has not yet been proven in phase 2 clinical trials (ClinicalTrials, 2024b). Another promising component, GB0139, an inhibitor of the pro-fibrotic galectin 3 (Hirani et al., 2021), failed in a phase 2 clinical trial as no improvement in the forced vital capacity of treated IPF patients was detected (ClinicalTrials, 2024a).

As an alternative strategy for novel target identification, integrative bioinformatics modeling aimed at further investigating the function of RNA-binding proteins (RBPs) in IPF pathogenesis. Here, analyses of bulk RNA-sequencing data of the lung tissue from IPF patients revealed a significant downregulation of the RBP Quaking (QKI) and a deregulation of several proteins in the QKI interactome (Stojanović et al., 2021).

In order to circumvent time- and cost-intensive *de novo* drug development, we cross-sectioned FDA-approved drugs listed in DrugBank (Wishart et al., 2018), with dysregulated factors in the QKI axis identified by Stojanović et al. (2021). With this drug-repurposing pipeline, we identified carvedilol, an inhibitor of β-adrenergic receptor (ADRB) families 1 and 2 and α-adrenergic receptor (ADRA) families 1 and 2 (Koshimizu et al., 2004; Mey et al., 1994; Nichols et al., 1991; O’Connell et al., 2013), which is clinically used for the treatment of various cardiovascular diseases [WHO Model List of Essential Medicines: 22nd List (2021)] as a promising candidate for IPF treatment. Upon determination of an effective non-toxic concentration, we validated the anti-fibrotic effects of carvedilol in multiple functional *in vitro* assays. With a translational perspective, we provide evidence that pro-fibrotic biomarker secretion of precision-cut lung slices (PCLSs) derived from healthy and fibrotic lungs recapitulates *in vivo* phenotypes and can be used for drug screening purposes.

## 2 Materials and methods

### 2.1 Cultivation of MRC-5 cells

Human embryonic lung fibroblasts (MRC-5) were cultivated in T-75 flasks (Sarstedt, Nümbrecht, Germany) in high-glucose Dulbecco’s modified Eagle’s medium (DMEM, 11965, Thermo Fisher Scientific, Waltham, MA, United States), supplemented with 1% penicillin–streptomycin (PenStrep, 15070, Thermo Fisher Scientific) and 10% fetal bovine serum (FBS, 10270, Thermo Fisher Scientific) at 37°C and 5% CO_2_. The medium was exchanged regularly, and cells were split weekly. For splitting, cells were washed with Dulbecco’s phosphate-buffered saline (PBS, Invitrogen, Waltham, MA, United States) and detached with 0.05% trypsin–EDTA solution (Invitrogen) for 5 min at 37°C. After incubation, the culture medium was added, and the suspension was centrifuged at 300 × *g* and 4°C for 5 min. The cells were resuspended in the culture medium, and viability and concentration were determined using the Countess II (Thermo Fisher Scientific) by mixing 10 μL of the cell suspension with an equal volume of 0.4% trypan blue stain (Invitrogen). For further cultivation, 300,000 cells were seeded per T-75 flask.

### 2.2 Treatment of cells

If not indicated otherwise, treatment of MRC-5 was initiated 24 h after seeding in the starvation medium (SM) with 0.1% FBS and 1% PenStrep. Cells were washed once with PBS beforehand. Exposure to carvedilol (C3993, SigmaAldrich) at a concentration of 15 µM was maintained for 48 h in the presence of either 5 ng/mL TGFβ (240-B, R&D Systems, Minneapolis, MN, United States) or respective vehicle [4 mM HCl containing 0.1% bovine serum albumin (810683, Sigma-Aldrich)].

### 2.3 Cellular viability

First, 7,500 MRC-5 were seeded per well in a 96-well plate and incubated for 24 h. After exposure to respective treatments for 48 h, the cytotoxicity was measured using the CytoTox 96^®^ Non-Radioactive Cytotoxicity Assay kit (Promega), according to the manufacturer’s instructions. A positive control was created by adding 1× lysis buffer for 30 min. The optical density at 490 nm was measured using the Synergy HT Reader (BioTek) after incubating the reaction mix that is light-protected for 20 min at room temperature (RT). Cellular metabolic activity was assessed by measuring the water-soluble tetrazolium salt (WST-1) turnover. To achieve this, the media was removed, and 100 µL per well of the Cell Proliferation Reagent WST-1 (Roche), diluted 1:10 in the culture medium, was added. Cells were incubated for approximately 90 min, and the absorbance at 450 nm (reference 630 nm) was detected using the Cytation 1 Cell Imager (BioTek).

### 2.4 Cell proliferation assay

MRC-5 cells were seeded at a concentration of 5,000 cells per well in a 96-well plate. Drug treatments were added, and cells were incubated for 24 h. 5-Bromo-2′-deoxyuridine (BrdU) labeling solution was diluted 1:100 in SM, and 10 µL/well was added to achieve a final concentration of 1:1,000. After incubating for 24 h, built-in BrdU was detected using the Cell Proliferation ELISA–BrdU kit (Roche), abiding by the manufacturer’s protocol. Absorbance at 370 nm was detected (reference 490 nm) using the Synergy HT Reader (BioTek).

### 2.5 Cell migration

Before seeding 15,000 MRC-5 per well, the 96-well plate was coated with 0.1% (weight per volume in water) gelatin for 30 min at 37°C. Cells were cultivated until layers reached confluence. MRC-5 were treated for 24 h in the medium containing 10% FBS and 1% PenStrep, and nuclei were stained with 5 μg/mL Hoechst 33342 for 15 min at 37°C. A 20-µL pipet tip (Sarstedt) was used for introducing a wound, and cells were washed with PBS once. Images in channels “DAPI” (*λ*_Excitation_ = 377 nm and *λ*_Emission_ = 447 nm) and Bright Field High Contrast mode were taken every 2 h using a Cytation 1 (BioTek). Cell nuclei were detected in the DAPI channel using Gen5, and relative covered wound areas were calculated using Fiji (Pijuan et al., 2019; Schmidt et al., 2024a).

### 2.6 Quantification of reactive oxygen species

The DCFDA/H_2_DCFDA–Cellular ROS assay (Abcam) was used to quantify the reactive oxygen species (ROS) levels. The ROS assay was conducted by seeding MRC-5 cells at a concentration of 10,000 cells per well in a 96-well plate. After 48 h, treatments were initiated and maintained for 48 h. The medium was discarded, and cells were washed once with PBS. Subsequently, 50 µL per well of 1x assay buffer containing 20 µM 2′,7′-dichlorodihydrofluorescein diacetate was added to the cells. After 45 min of incubation at 37°C, the staining solution was removed, and cells were washed with PBS. Respective treatments were added to the cells in 1x assay buffer with or without 500 µM H_2_O_2_, and the fluorescence intensity at 535 nm (*λ*_Excitation_ = 485 nm) was quantified using a Cytation 1 (BioTek) for 6 h every 20 min.

### 2.7 Quantification of mRNA expression

MRC-5 cells were seeded at a concentration of 50,000 cells per well in a 24-well plate. After treatment, the media was discarded, and cells (four wells per condition) were harvested in 1 mL QIAzol Lysis Reagent (Qiagen) per condition. For RNA extraction, 200 µL chloroform was added, and the solution was vortexed for 15 s and incubated at RT for 5 min. After centrifuging at 12,000 × g and RT for 5 min, 500 µL of the aqueous phase was mixed with 500 µL isopropanol and incubated for 5 min on ice. After centrifugation for 10 min at 12,000 × g and 4°C, the pellet was washed with 75% (volume per volume in water) ethanol and centrifuged at 12,000 × g and 4°C for 10 min. After repeating the washing step once, the supernatant was removed, and the pellet was dried for approximately 15 min at RT. The pellet was dissolved in 20 µL RNase-free water (Biozym) and incubated for 3 min on ice. The concentration and purity were quantified using a Synergy HT Reader (BioTek). If necessary, samples were processed using the RNase-free DNase Set (Qiagen) to remove DNA contaminants. A reaction contained 0.0164 Kunitz Units/μL of DNase I in Buffer RDD and 0.533 U/μL of RNasin^®^ ribonuclease inhibitor. After incubating at 37°C for 30 min, 1.225 mM EDTA (15575020, Invitrogen) was added. The solution was incubated for 5 min at 65°C. Gene expression was analyzed by performing complementary DNA (cDNA) synthesis using the Biozym cDNA Synthesis Kit (331470, Biozym, Hessisch Oldendorf, Germany) with Oligo-(dT) primers, followed by real-time quantitative polymerase chain reaction (RT-qPCR) using the iQ™ SYBR^®^ Green Supermix (170888, Bio-Rad, Hercules, CA, United States), both according to the manufacturer’s instructions. Primer pairs (see Table 1) were applied at concentrations of 0.5 µM; ROX reference dye (from ABsolute Blue QPCR Mix, AB4136B, Thermo Fisher Scientific) and Precision Blue™ (172555, Bio-Rad) were added to qPCR mixes at volumetric ratios of 1:10,000 and 1:400, respectively. The final concentration of cDNA was 2 ng/μL. qPCRs were performed in 384-well plates using a Quant Studio 7 Flex system with QuantStudio™ Real-Time PCR software (both ABI, Waltham, MA, United States). Primer sequences are found in Table 1.

**TABLE 1:** Sequences of primer pairs used in this project.

Target	Forward sequence (5′ to 3′)	Reverse sequence (3′ to 5′)
*ACTA2*	CCT​GAC​TGA​GCG​TGG​CTA​TT	GAT​GAA​GGA​TGG​CTG​GAA​CA
*ACTC1*	AGC​CCT​CCT​TCA​TTG​GTA​TGG	CGC​TCA​GGG​GGA​GCA​ATA​AT
*ADRA1A*	CTT​AGT​CAT​GCC​CAT​TGG​GTC​TTT​C	TTT​ACT​TCT​CAC​CCG​GGC​TGT
*ADRA1B*	TCC​CTC​TGG​CGG​TCA​TTC​TA	GGA​GAA​CAA​GGA​GCC​AAG​CG
*ADRA2C*	GTA​CAA​CCT​GAA​GCG​CAC​AC	GGA​TGT​ACC​AGG​TCT​CGT​CG
*ADRB1*	GGA​ATC​CAA​GGT​GTA​GGG​CC	TTC​AGA​CGA​GGA​TTG​TGG​GC
*ADRB2*	AAG​CCC​TCA​AGA​CGT​TAG​GC	AGG​CAC​AGT​ACC​TTG​ATG​GC
*ATP2A2*	ATG​GGG​CTC​CAA​CGA​GTT​AC	CAC​CTT​CTT​CAA​ACC​AAG​CCA
*CDH2*	AAG​AGA​CCC​AGG​AAA​AGT​GGC	TCT​GCT​GAC​TCC​TTC​ACT​GAC​T
*COL1A1*	ACG​AAG​ACA​TCC​CAC​CAA​TC	CTT​GGT​CGG​TGG​GTG​ACT​CT
*CTGF*	GTGTGCACCGCCAAAGAT	GTG​TCT​TCC​AGT​CGG​TAA​GC
*GUSB*	GAC​ACC​CAC​CAC​CTA​CAT​CG	CTT​AAG​TTG​GCC​CTG​GGT​CC
*ITPR1*	AGT​TCA​AAA​GCC​CTG​TGG​GAG	GCA​TTC​TTC​CTC​AAA​GTC​AGG​GT
*POSTN*	TAG​TCG​TAT​CAG​GGG​TCG​GG	TGG​GCA​GCC​TTT​CAT​TCC​TT
*VCAM1*	TGA​GGA​GTG​AGG​GGA​CCA​ATT	TGG​ATC​TCT​AGG​GAA​TGA​GTA​GAG

*ACTA2*, alpha smooth muscle actin 2; *ACTC1*, cardiac muscle alpha actin 1; *ADRA*, alpha adrenergic receptor; *ADRB*, beta adrenergic receptor; *ATP2A2*, sarcoplasmic/endoplasmic reticulum calcium ATPase 2; *CDH2*, cadherin 2; *COL1A1*, collagen type 1 alpha 1; *CTGF*, connective tissue growth factor; *GUSB*, beta glucuronidase; *ITPR1*, inositol 1,4,5-trisphosphate receptor type 1; *POSTN*, periostin; *VCAM1*, vascular cell adhesion molecule 1.

### 2.8 Proteomics

Proteomics analyses were performed as described in a previous publication (Schmidt et al., 2024b). In brief, 30 µg of protein isolated from treated MRC-5 cells was separated on a gradient gel and subsequently analyzed by liquid chromatography coupled with Orbitrap mass spectrometry, resulting in the identification of 4,386 proteins. Of those, 1,955 proteins fulfilling respective criteria were further analyzed for differential expression among groups. Overrepresentation analysis (ORA) of Gene Ontology (GO) terms among significantly regulated proteins (ǀintensity differenceǀ ≥1 and adjusted p-value ≤0.05) was performed using WebGestalt (Liao et al., 2019). Proteomics data are provided in Supplementary Table S1.

### 2.9 Precision-cut lung slices

PCLSs were generated from human lung lobes of female and male patients with an average age of 63 ± 5.6 years undergoing lung resection for cancer or transplantation for end-stage fibrotic interstitial lung disease (Table 2). Non-diseased tissue from tumor resection or fibrotic tissue was processed immediately after resection. In brief, a lung lobe was inflated with 2% agarose/medium solution. After polymerization, tissue cores with a diameter of 8 mm were cut into thin tissue slices with a thickness of 300 µm (Neuhaus et al., 2018). PCLSs were cultivated in a 24-well plate with 250 µL F-12/DMEM (11039-021, Gibco) with 1% PenStrep per PCLS and well for approximately 16 h before treatment. PCLSs were exposed for 48 h to carvedilol (C3993, SigmaAldrich) at concentrations of 15 μM, 1.5 µM, and 0.15 µM. In addition, unaffected cancer resections were pro-fibrotically stimulated by the application of 5 ng/mL TGFβ. (240-B, R&D Systems, Minneapolis, MN, United States).

**TABLE 2:** Demographics of lung tissue donors.

Sex	Age	Diagnosis
F	69	Tumor (adenocarcinoma)
F	68	Tumor (adenocarcinoma)
F	64	Tumor (adenocarcinoma)
F	58	End-stage fibrosis (due to EAA)
M	65	End-stage fibrosis (due to EAA)
M	55	End-stage fibrosis (due to EAA)

*EAA*, extrinsic allergic alveolitis.

### 2.10 Ethics statement

The utilization of human lung tissue in experiments was approved by the ethics committee of Hannover Medical School and complies with the World Medical Association’s Code of Ethics (updated on 04/22/2015, identified as number 2701-2015). All patients provided written informed consent for the use of their organ tissue in research.

### 2.11 miR-21 abundancy in the supernatant *ex vivo*

After cultivation of PCLS, the supernatants were collected, and isolation of miRNA was done by using the miRNeasy Serum/Plasma Advanced Kit (217204, Qiagen), along with spike-in of synthetic cel-miR-39-3p, according to the manufacturer’s protocol. miRNAs were diluted in nuclease-free water, and reverse transcriptions were performed using the TaqMan™ MicroRNA Reverse Transcription Kit (4366597, Applied Biosystems). qPCRs of miR-21 and cel-miR-39-3p for expression level normalization were performed in 384-well plates with respective TaqMan™ Assays (hsa-miR-21, #000397; cel-miR-39-3p, #000200) using a Quant Studio 7 Flex system. Data were analyzed using QuantStudio™ Real-Time PCR software.

### 2.12 Statistical analysis

Statistical analyses were performed using GraphPad Prism 9. Normal distribution of data was proven using the Shapiro–Wilk test. Unless stated otherwise, statistical analysis was performed using two-way analysis of variance (ANOVA) and *post hoc* pairwise comparisons corrected according to Šídák’s method. Graphs depict means ± standard deviations. (Adjusted) *P* values less than or equal to 0.05 were considered statistically significant.

## 3 Results

### 3.1 Identification of carvedilol as a putative anti-IPF treatment

**FIGURE 1:**
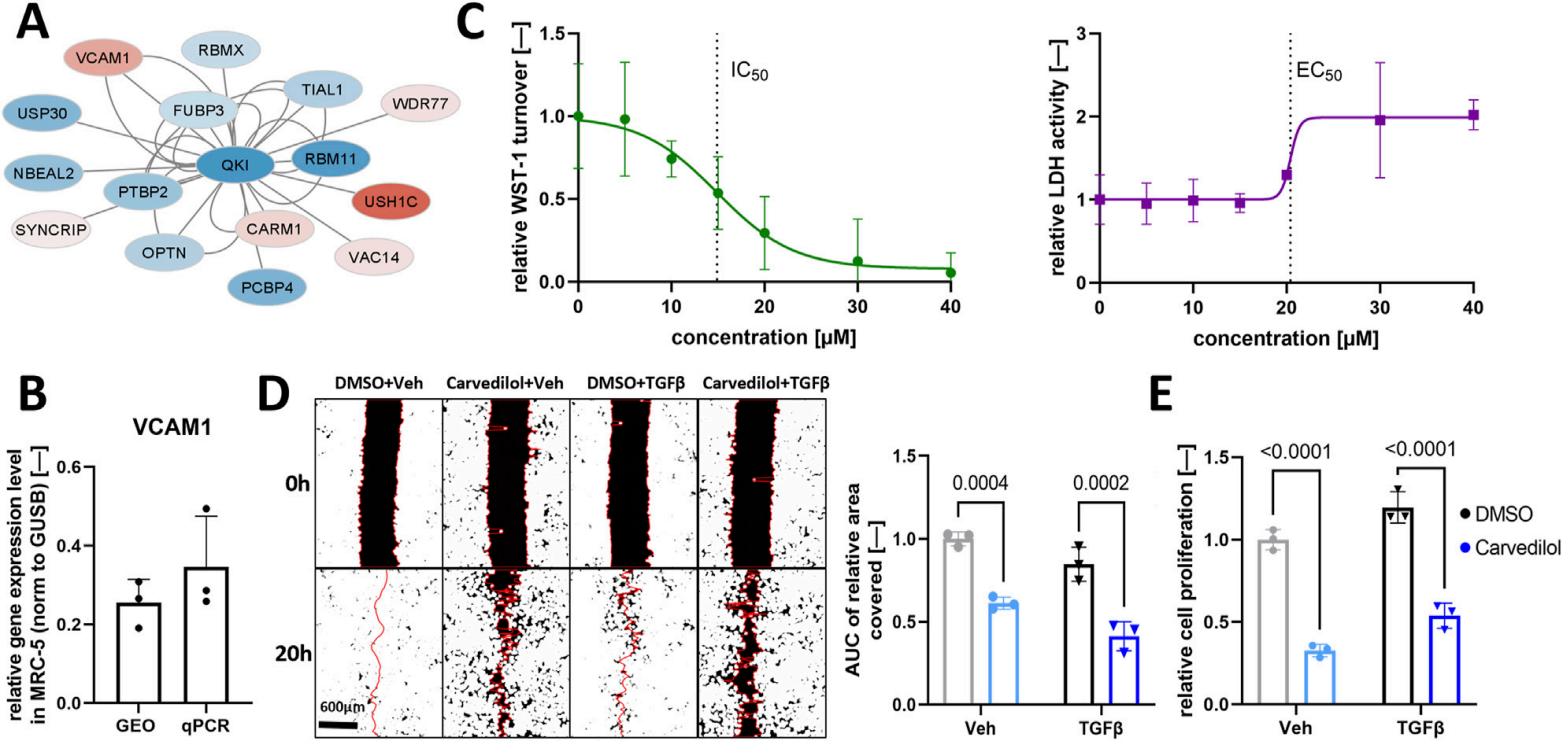
Carvedilol treatment of pulmonary fibroblasts inhibits functional pro-fibrotic characteristics. **(A)** Regulation of the interactome of Quaking (QKI) in idiopathic pulmonary fibrosis. Blue and red fillings indicate downregulation and upregulation, respectively. Data are obtained from Stojanović et al., 2021. **(B)** *VCAM1* expression normalized (norm.) to β-glucoronidase (GUSB) in MRC-5 with Gene Expression Omnibus (GEO) datasets and real-time quantitative polymerase chain reaction (qPCR) analysis (*n* = 3). **(C)** Dose-finding experiments in MRC-5 with water-soluble tetrazolium salt-1 (WST-1) assay (left) and CytoTox 96^®^ Assay (right) after 48 h of carvedilol treatment. Lines depict the four-parameter logistic model used to determine the half-maximal inhibitory concentration (IC_50_) and half-maximal effective concentration (EC_50_) (*n* = 3). **(D)** Representative images of the scratch wound assay of respectively treated MRC-5 (left) and areas under curve (AUC) of relative wound areas covered over time normalized to dimethyl sulfoxide (DMSO)/vehicle (Veh) (right) (*n* = 3). Two-way ANOVA *p* (interaction) = 0.5990. **(E)** Relative cell proliferation rates quantified using the Cell Proliferation ELISA–BrdU kit in respectively treated MRC-5 (*n* = 3). Two-way ANOVA *p* (interaction) = 0.8508. TGFβ, transforming growth factor β.

Previously, data mining of transcriptomics data of the lung tissue of IPF patients revealed deregulation in the QKI interactome (Stojanović et al., 2021). *In silico*-based screening for druggable targets highlighted FDA-approved carvedilol as an inhibitor of vascular cell adhesion molecule 1 (VCAM1) synthesis as a promising candidate for IPF treatment (Figure 1A). We investigated the potential of carvedilol in human embryonal lung fibroblasts MRC-5 since fibroblast recruitment and differentiation are key players in IPF pathogenesis (Richeldi et al., 2017). *VCAM 1* expression in MRC-5 was validated by analyzing independent gene expression datasets (NCBI-GEO, 2024a; NCBI-GEO, 2024b; NCBI-GEO, 2024c) and by performing qPCR analyses (Figure 1B), underlining the rationale of *in silico* prediction. Viability screening in MRC-5 cells combining WST-1 and LDH assays was performed to determine effective carvedilol concentrations and the toxic range. A half-maximal inhibitory concentration (IC_50_) of approximately 15 µM and a half-maximal effective concentration (EC_50_) of 20.41 µM were identified (Figure 1C), prompting the utilization of 15 µM as a working concentration in subsequent experiments.

### 3.2 Carvedilol represses functional characteristics of activated fibroblasts

We investigated the impact of carvedilol on hallmarks of fibrogenesis that ultimately lead to damage of alveoli and lung failure in IPF (Martinez et al., 2017). In line, the fibroblast-to-myofibroblast transition process was induced by TGFβ application. Carvedilol treatment resulted in significantly decreased wound closure in TGFβ-stimulated and non-stimulated MRC-5 compared to the DMSO control group in an artificial wound healing assay monitored for 24 h (Figure 1D). Next, the proliferative capacity of MRC-5 was examined by detecting BrdU incorporation in newly synthesized DNA and demonstrated significantly reduced proliferation after carvedilol treatment (Figure 1E).

### 3.3 Carvedilol impairs pro-fibrotic gene expression signatures

**FIGURE 2:**
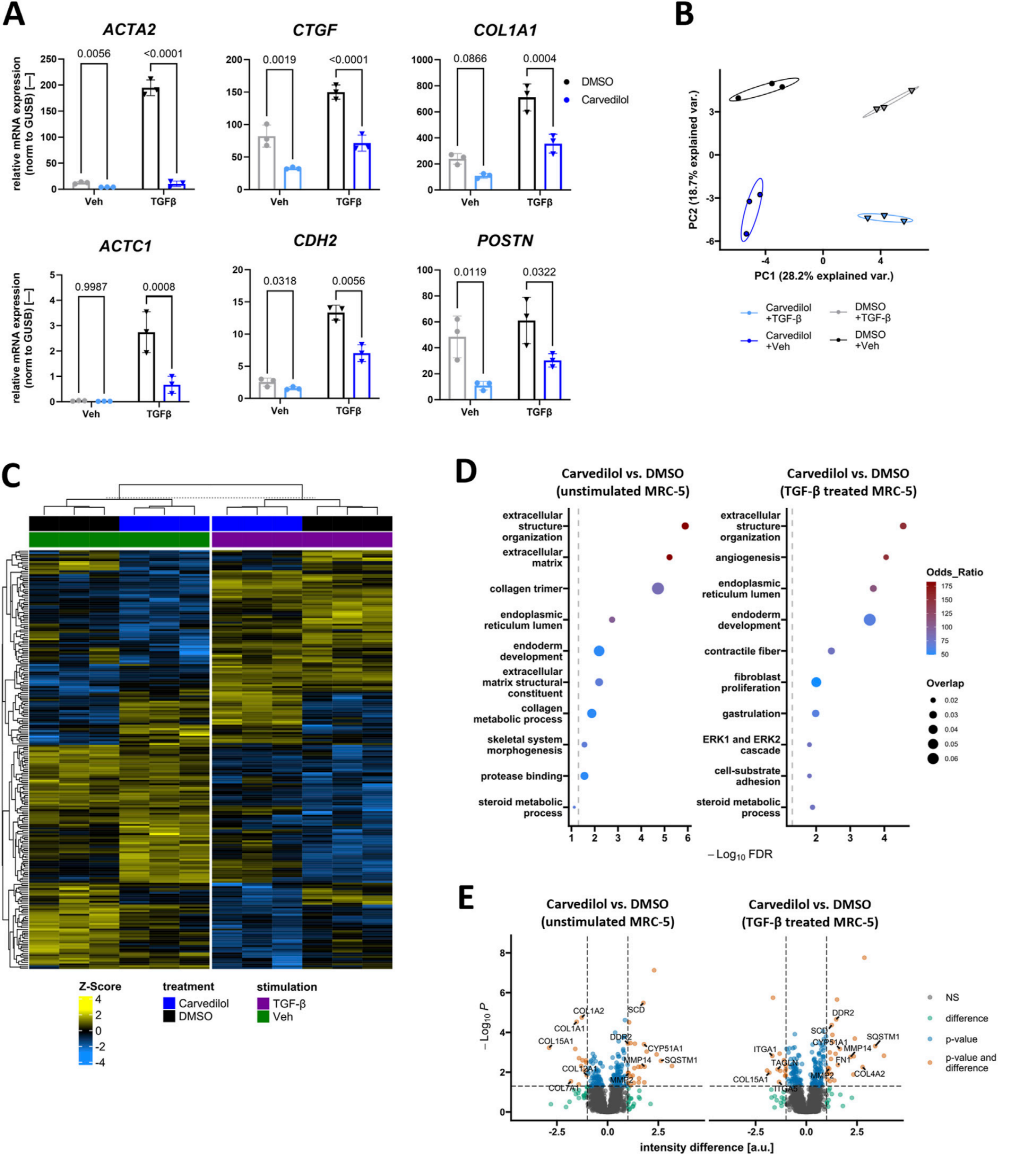
Carvedilol represses pro-fibrotic signaling in lung fibroblasts. **(A)** Relative mRNA expression of fibrosis-associated genes in MRC-5 treated with dimethyl sulfoxide (DMSO) control or carvedilol and stimulated with transforming growth factor β (TGFβ) or vehicle (Veh) control. α-smooth muscle actin (*ACTA2*), periostin (*POSTN*), cadherin 2 (*CDH2*), and cardiac muscle α-actin (*ACTC1*) data were log-transformed before statistical calculations. CTGF, connective tissue growth factor; COL, collagen. Two-way ANOVA *p* (interaction)-values: *ACTA2* < 0.001, *CTGF* < 0.0662, *COL1A1* 0.0196, *POSTN* 0.6455, *CDH2* 0.0011, and *ACTC1* 0.0035. **(B)** Principal component (PC) analysis of proteomics data from MRC-5 exposed to different treatments. Var, variance. **(C)** Heatmap showing protein Z-scores of significantly regulated (|difference| ≥ 1, adj. p-value ≤ 0.05) candidates in the dataset. **(D)** Analysis of significant regulation of proteins by carvedilol in Veh (left) or TGFβ-stimulated MRC-5 (right) for overrepresented Gene Ontology terms. FDR, false discovery rate. **(E)** Volcano plots highlighting the differential expression of proteins comparing carvedilol and DMSO groups. Annotated candidates are related to extracellular matrix remodeling and other fibrosis-associated aspects. NS, not significant; a.u., arbitrary units. All analyses were conducted with *n* = 3.

To confirm the disclosed morphological effects of carvedilol on molecular levels, we compared expressed mRNA levels of selected markers crucially involved in fibrogenesis (Hillsley et al., 2022) in non-stimulated and TGFβ-stimulated MRC-5 exposed to carvedilol. As expected, TGFβ stimulation increased gene expressions of the known fibrosis markers alpha smooth muscle actin 2 (*ACTA2*) and connective tissue growth factor (*CTGF*), the migration-associated factors cardiac muscle alpha actin 1 (*ACTC1*) and cadherin 2 (*CDH2*), as well as the ECM remodeling marker collagen type 1 alpha 1 (*COL1A1*). Of great importance, carvedilol counteracted this stimulation and additionally repressed the expressions of *ACTA2*, *CTGF*, *COL1A1*, and *CDH2* in non-stimulated MRC-5. In line with this, the migration-associated factor periostin (*POSTN*) was repressed in non-stimulated (and to a lesser extend in TGFβ-stimulated) MRC-5 after carvedilol treatment (Figure 2A). To investigate whether the anti-fibrotic impacts of carvedilol translate to protein levels, we performed unbiased proteomics analyses of MRC-5 exposed to carvedilol in the presence or absence of TGFβ stimulation. Dimensional reduction in the data by principal component analysis could clearly outline the profound impacts of TGFβ and carvedilol as investigated groups separated well from each other (Figure 2B). In total, 228 significantly regulated (|difference| ≥ 1, adj. *p*-value ≤ 0.05 in any comparison) proteins could be identified in the dataset. As shown in the heatmap, Euclidean distance-based clustering of samples underlined strong effects of TGFβ and carvedilol on the MRC-5 proteome (Figure 2C). Analyses of significantly regulated candidates by carvedilol for overrepresented GO terms highlighted a strong influence of ECM-associated factors (Figure 2D). Of note, many collagen types were downregulated by carvedilol, whereas ECM-degrading enzymes such as MMP2 and MMP14 were consistently upregulated in both stimulated and non-stimulated MRC-5 (Figure 2E). As expected, comparing significantly regulated proteins by carvedilol in TGFβ-stimulated MRC5 with those regulated by TGFβ stimulation alone (Supplementary Figure S1A) highlighted several candidates that were adversely regulated, with many more showing statistical significance in one group and respective trends in the other (Supplementary Figure S1B). In line, GO term overrepresentation analysis in TGFβ-induced proteome alterations listed various ECM-related pathways, underlining the countering effects of carvedilol (Supplementary Figure S1C).

### 3.4 Investigation of potential molecular mechanisms underlying the anti-fibrotic effects of carvedilol

**FIGURE 3:**
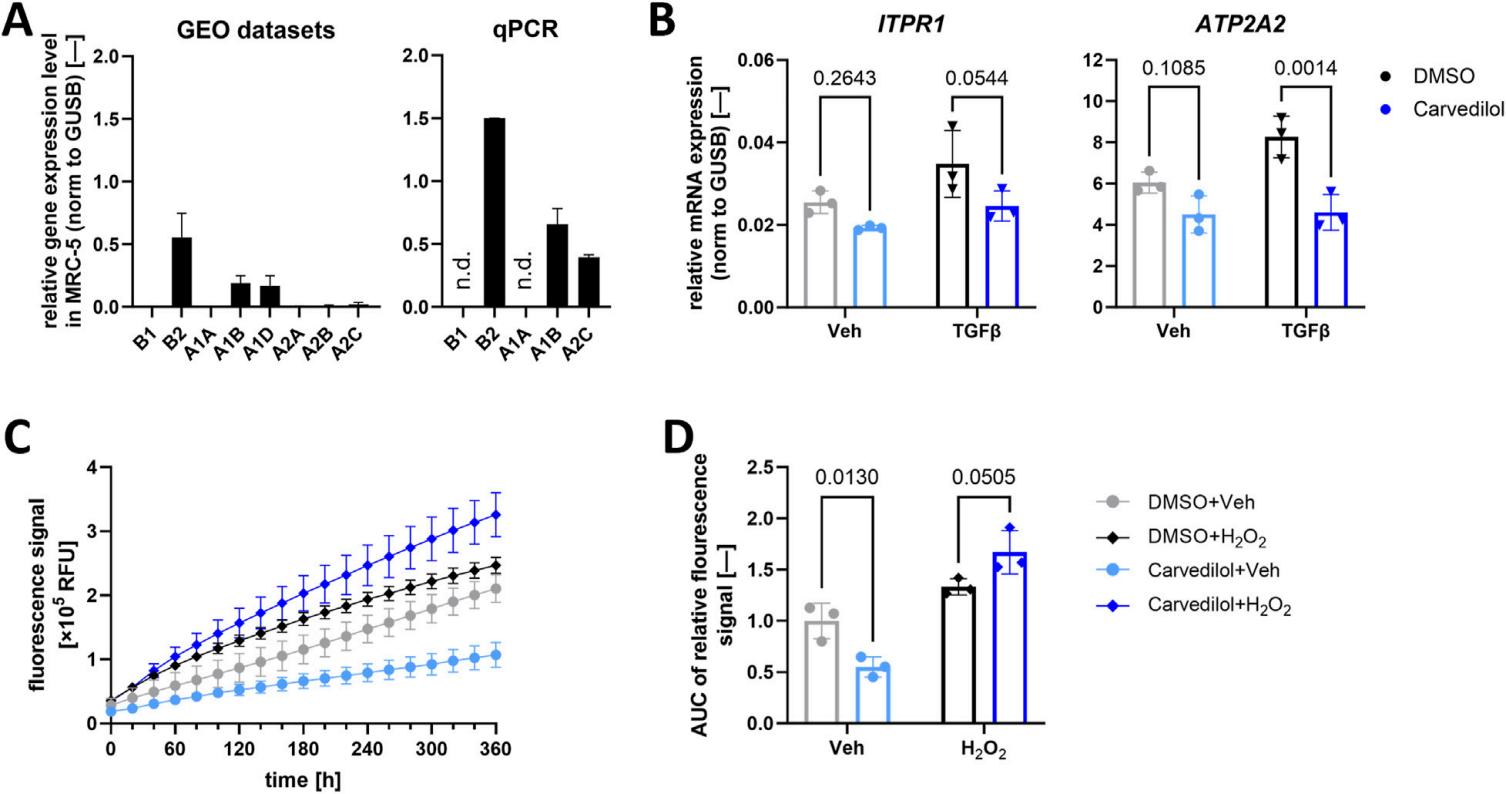
Carvedilol influences downstream α- and β-adrenoreceptor signaling. **(A)** Relative mRNA expression of α- and β-adrenoreceptor subtypes in MRC-5 in three independent Gene Expression Omnibus (GEO) datasets (left) and quantified via quantitative polymerase chain reaction (qPCR) analysis (right). N.d., not detectable. **(B)** Relative mRNA expression of inositol 1,4,5-trisphosphate receptor type 1 (*ITPR1*) and sarcoplasmic/endoplasmic reticulum calcium ATPase 2 (*ATP2A2*) normalized (norm.) to β-glucoronidase (*GUSB*). Lung fibroblasts were treated with dimethyl sulfoxide (DMSO) control or carvedilol and pro-fibrotic stimulation with transforming growth factor β (TGFβ) or vehicle (Veh) control. Two-way ANOVA *p* (interaction)-values: *ITPR* 0.4746 and *ATP2A2* 0.0618. **(C)** Representative reactive oxygen species (ROS) levels measured via recording the fluorescence intensity of MRC-5 after DCFDA staining over time. **(D)** Areas under curves (AUCs) of measured ROS levels normalized to DMSO/Veh. Two-way ANOVA *p* (interaction) < 0.0020. All analyses were conducted with *n* = 3.

Proofing established anti-fibrotic effects of carvedilol on a functional and transcriptional level in MRC-5, we then focused on potential underlying molecular modes of action. It is widely known that carvedilol primarily functions as a non-selective α- and β-adrenoreceptor antagonist targeting ADRB1, ADRB2, ADRA1, and ADRA2 families (Wu et al., 2021), some of which are not ubiquitously expressed. For this reason, we assessed the presence of respective adrenoreceptor families in MRC-5. Gene expression profiling of MRC-5 was performed by analyzing three independent GEO datasets (NCBI-GEO, 2024a; NCBI-GEO, 2024b; NCBI-GEO, 2024c), suggesting an abundance of *ADRB2*, *ADRA1B*, *ADRA1D*, and *ADRA2C*. Of those, *ADRB2*, *ABRA1B*, and *ADRA2C* could be validated in qPCR analyses (Figure 3A). ADRs are a class of G-protein-coupled receptors with a signaling cascade ultimately altering intracellular Ca^2+^ (Fernandez-Tenorio and Niggli, 2018; Wu et al., 2021), which, as a secondary messenger, has been described to profoundly influence pro-fibrotic events including fibroblast activation, migration, and proliferation (Roach and Bradding, 2020). Thus, hypothesizing that carvedilol acts in an anti-fibrotic manner through ADR inhibition and subsequently altered Ca^2+^ homeostasis, we evaluated the expressions of key Ca^2+^-regulating factors in (treated) MRC-5. In line with this, carvedilol reduced the gene expressions of inositol 1,4,5-trisphosphate receptor type 1 (*ITPR1*) and sarcoplasmic/endoplasmic reticulum calcium ATPase 2 (*ATP2A2*) encoding for the SERCA2 protein in TGFβ-stimulated lung fibroblasts (Figure 3B). Ca^2+^-signaling pathways engage in crosstalk with various other cellular signaling networks, including the generation of ROS. These interactions play a critical role in regulating numerous cellular functions (Görlach et al., 2015). Therefore, the potential antioxidant effects of carvedilol were investigated in human lung fibroblasts. Analyses of ROS levels over time revealed a significant decrease in ROS levels in MRC-5 during carvedilol treatment. On the contrary, upon oxidative stress induced with H_2_O_2_, MRC-5 tended to generate more ROS when exposed to carvedilol (Figures 3C, D).

### 3.5 PCLS model mimics pro-fibrotic biomarker miR-21 secretion in patients

**FIGURE 4:**
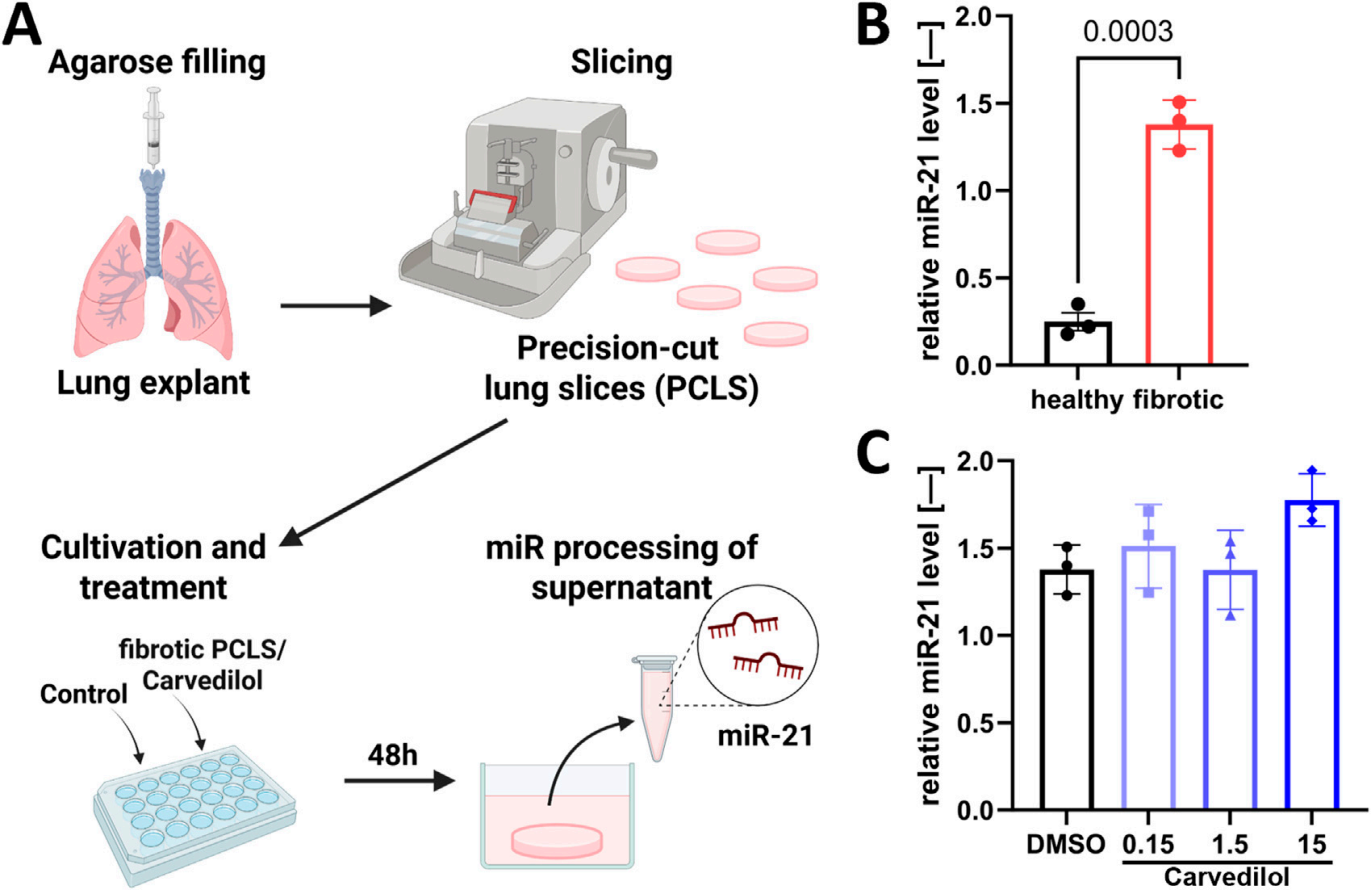
The PCLS model mimics the pro-fibrotic biomarker micro RNA (miR)-21 secretion in patients. **(A)** PCLSs were cultivated and treated for 48 h with subsequent detection of secreted miR-21 in supernatants. **(B)** miR-21 levels in supernatants from healthy and fibrotic PCLSs. Statistical analyses were performed using unpaired t-test. **(C)** Relative miR-21 levels in the supernatant (norm to cel-miR-39) of fibrotic PCLSs after carvedilol treatment [dimethyl sulfoxide (DMSO) from **(B)** “fibrotic”]. Statistical analyses were performed with one-way ANOVA with *post hoc* Dunnett’s multiple comparison test. Analyses were conducted with *n* = 3 donors of fibrotic tissue and *n* = 3 donors of healthy tissue.

Although adherent lung fibroblast culture systems are well-established and can represent cellular mechanisms, they show limitations in modeling native lung tissue for multiple reasons, including a lack of essential intercellular and cell–ECM interactions. To overcome this translational gap, we used *ex vivo* human PCLSs (Pijuan et al., 2019; Sailer et al., 2023). Avoiding the complex and error-prone RNA isolation procedures from cultured PCLSs (Stegmayr et al., 2021), we focused on assessing parameters from culture supernatants (Figure 4A). Comparing non-fibrotic and fibrotic PCLSs, we could demonstrate that secreted miR-21 levels serve as a robust biomarker for the fibrotic state of the *ex vivo* cultured tissue (Figure 4B). However, carvedilol treatment on fibrotic PCLSs for 48 h did not influence the abundance of miR-21 in the supernatant (Figure 4C).

## 4 Discussion

By combining *in silico* and experimental approaches, we herein identified the cardiac drug carvedilol as a potential novel treatment option for IPF. Drug screening on the deregulated Quaking interactome in IPF patients highlighted carvedilol as an inhibitor of VCAM1 synthesis (Chen et al., 2004). VCAM1 is elevated in fibrotic lungs (Stojanović et al., 2021) and plays a crucial role in the progression of inflammatory processes. Interestingly, sustained high expression of VCAM1 in fibroblasts is associated with elevated collagen production (Sima et al., 2022), as well as promotion of migratory and proliferative behaviors of several lung cancer cell lines (Zhou et al., 2020). We could successfully validate that *VCAM1* is abundant in the used MRC5 lung fibroblasts, underlining these cells *in vitro* as an apt model for further exploring the anti-fibrotic potential of carvedilol.

Prior to investigations of carvedilol’s impact on intrinsic cellular aspects relevant to fibrosis, we determined both effective and non-toxic concentrations. The estimated concentrations lie within the range of previously reported concentrations for other cell types, such as cardiomyocytes (Seo et al., 2023), hepatic stellate cells (Meng et al., 2018), or T cells (Yang et al., 2003). As expected, the subsequently applied concentration of 15 µM does not accurately reflect the *in vivo* situation, where maximum plasma concentrations remain below 1 µM (McPhillips et al., 1988). However, the dosage comparability to preclinical applications in the cardiovascular context encourages the pursuit of repurposing for pulmonary pathologies.

Using human lung fibroblasts *in vitro*, we could confirm ameliorating effects on several aspects of fibroblast activation on transcriptional, translational, and functional levels. The transition of quiescent fibroblasts to activated myofibroblasts, the decisive step in fibrosis progression, encompasses a broad set of functional changes including heightened proliferative and migratory activities of the cells (Richeldi et al., 2017). Interestingly, both aspects were significantly reduced by carvedilol, underlining the anti-fibrotic potential. Even though the cultivation of diverse fibroblast cell lines and primary cells on plastic surfaces immediately activates these cells to a certain degree (Jensen and Teng, 2020), we additionally introduced highly pro-fibrotic TGFβ stimulation. Although TGFβ accelerated the proliferation of MRC5, wound closure rates were unaltered. In both assays, however, the impact of carvedilol persevered despite the additional stimulation. In addition to functional assessment, we monitored the expressions of key fibrosis marker genes upon *in vitro* treatment. Downregulation of *ACTA2*, *CTGF*, and *CDH2* in both stimulated and unstimulated MRC5 treated with carvedilol highlighted the broad anti-fibrotic action of this drug and validated the results from *in vitro* assays.

Adding to the results gained from functional assays and transcriptional analyses that only covered selected aspects of fibrotic processes, we performed global proteomics analyses to obtain a more comprehensive overview of the anti-fibrotic action of carvedilol and shed light on the potential underlying molecular events. In line with the significant reduction in *COL1A1* mRNA expression, a strong carvedilol-induced dysregulation of proteins involved in ECM production and remodeling was apparent. Deposition and organization of ECM components, primarily by fibroblasts, is a crucial player in fibrosis development and progression in many organs, including the lungs, as the functional parenchyma is replaced by a stiff scar hampering oxygen uptake (Richeldi et al., 2017). This profound inhibition of adverse remodeling processes by carvedilol further highlights the potential of this drug for the treatment of IPF. However, from the current data, it is not possible to infer whether carvedilol application would actually reverse or rather just slow down IPF progression, comparable to nintedanib and pirfenidone.

Interestingly, proteomics data also indicated a change in processes related to the endoplasmic reticulum (ER) elicited by carvedilol treatment. It is widely known that, among other characteristics, the ER is the main responsible organelle for intracellular Ca^2+^ storage and homeostasis (Görlach et al., 2015; Roach and Bradding, 2020). As previously outlined, downstream signaling cascades of ADRs, the known targets of carvedilol, heavily involve Ca^2+^ as a second messenger (Fernandez-Tenorio and Niggli, 2018; Wu et al., 2021). Therefore, we investigated whether the observed anti-fibrotic effects may be rooted in an alteration of cellular Ca^2+^ homeostasis ensuing from ADR blockage. In support of this notion, we could validate the expressions of *ADRB2* and *ADRA1B* subtypes in MRC5 cells and showed an influence of carvedilol on the expression of Ca^2+^-handling genes. An aberrant cellular Ca^2+^ balance has been described to profoundly disturb mitochondrial function (Görlach et al., 2015), which can, in turn, result in higher ROS abundance. Carvedilol has dose-dependent antioxidant properties by inhibiting the formation of ROS in human neutrophils (Yue et al., 1992). H_2_DCFDA assays showed a reduction in intracellular ROS levels by carvedilol in unchallenged cells, while treated cells responded slightly worse to H_2_O_2_ exposure. This underlines the hypothesis of the ADR-Ca^2+^-axis as an underlying molecular mode of action of carvedilol in lung fibroblasts. The investigation on well-characterized fetal MRC5 ensures valuable insights into initial research on fibrosis. It has to be stated though that the TGFβ-MRC5-model used in this work is highly artificial and simplistic, thus not covering certain IPF-relevant aspects. As such, while preserving the benefits of cell culture models, future studies could utilize primary diseased lung fibroblasts to overcome certain limitations of MRC5-based models, e.g., the absence of age-associated senescence, a central player in IPF pathophysiology (Schafer et al., 2017). Nonetheless, more complex processes, such as disease-relevant ECM remodeling, cannot be captured in 2D *in vitro* setups and require more advanced models mimicking the *in vivo* complexity. Consequently, to draw meaningful conclusions in that regard, additional experiments are indispensable.

The translation from *in vitro* 2D cultures to *in vivo* remains a major challenge in pharmacological research (Kreutzer et al., 2022). Especially in the context of IPF, animal models such as bleomycin-treated mice certainly fail to overcome this problem as they lack specific pathophysiological features of IPF observed in humans (Ye et al., 2023). In line with the 3R principle, PCLSs generated from human non-fibrotic or fibrotic lung samples have emerged as valuable tools for preclinical research and might help facilitate translation from *in vitro* data to the clinical level. MiR-21 has been well-characterized as a pro-fibrotic factor especially in cardiac diseases (Lorenzen et al., 2015; Ramanujam et al., 2016; Thum et al., 2008) and also influences fibrosis in multiple other organs (Chau et al., 2012; Zhang et al., 2015). Of note, elevated miR-21 levels have been reported in bleomycin mouse model lungs, as well as the pulmonary tissue of IPF patients (Liu et al., 2010). For the first time, we could demonstrate in this work that such a phenotype is accurately represented in the supernatant of fibrotic lung-derived PCLSs cultivated for 48 h compared to healthy controls. Although clinical assessment of biomarkers for interstitial lung disease progression from liquid biopsies (blood or sputum) cannot fully be transferred to *ex vivo* tissue cultures, we here describe an easy and reliable alternative for assessing the fibrotic state of cultivated PCLSs. In contrast to the previously described effects in the 2D *in vitro* setting, addition of carvedilol to fibrotic PCLSs could not counteract the excessive secretion of miR-21. One possible explanation for this discrepancy could be an insufficient exposure time for the highly fibrotic human tissue. Extending the culture time of PCLSs, e.g., through the implementation of a microfluidic system or bioreactor technology, could, therefore, improve fibrosis modeling in the context of preclinical drug development (Leung et al., 2022; Paish et al., 2019). More insights into the impact of carvedilol on molecular signaling *ex vivo* is needed in future studies.

**FIGURE 5:**
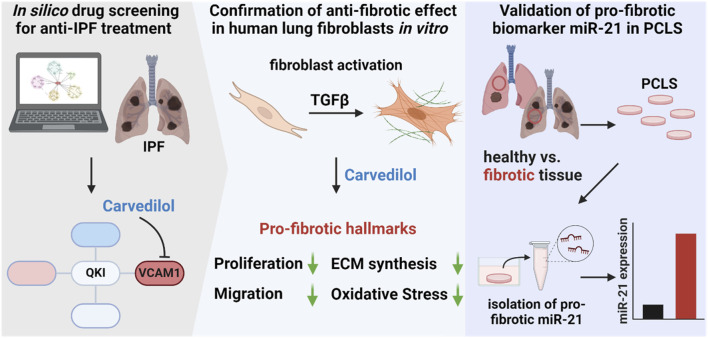
Identification of carvedilol as potential idiopathic pulmonary fibrosis (IPF) treatment option and validation of pro-fibrotic biomarker miR-21 in *ex vivo* precision-cut lung slices (PCLSs). *In silico* analysis of dysregulated pathways in IPF and screening for interfering small molecules highlighted carvedilol. The anti-fibrotic effect was confirmed in human lung fibroblasts where carvedilol inhibited cell proliferation, reduced migratory activity, decreased extracellular matrix synthesis, and repressed reactive oxygen species levels. Additionally, miR-21 was validated as a secreted pro-fibrotic biomarker in the *ex vivo* PCLS model.

Collectively, we identified carvedilol as a potential drug-repurposing approach for the treatment of IPF. Our findings revealed a reduction in migration and proliferation in human lung fibroblasts, as well as a repression of the expression of pro-fibrotic marker genes and a strong influence on ECM-associated factors on the translational level. From a mechanistic standpoint, ADR blockage by carvedilol might exert an influence on Ca^2+^ signaling, ultimately leading to a reduction in ROS generation in fibroblasts. At the biomarker level, we considered the pro-fibrotic miR-21 secretion in *ex vivo* PCLSs from healthy and fibrotic tissues for the first time, which ultimately aligns with the patient’s situation representing enhanced miR-21 abundancy in the human fibrotic lungs (Figure 5). Strong anti-fibrotic features of carvedilol underscore the potential for a possible beneficial therapy for IPF patients in early stages of fibrosis. Our drug repurposing strategy is thus an elegant way to introduce safe drugs for its therapeutic use in the context of lung remodeling, offering alternative clinical care to gold standards nintedanib and pirfenidone.

## Data Availability

Publicly available datasets were analyzed in this study. This data can be found here: https://www.ncbi.nlm.nih.gov/geo/query/acc.cgi?acc=GSE118693, https://www.ncbi.nlm.nih.gov/geo/query/acc.cgi?acc=GSE117811, https://www.ncbi.nlm.nih.gov/geo/query/acc.cgi?acc=GSE117814. The original contributions presented in the study are included in the article/supplementary material, further inquiries can be directed to the corresponding author/s.
