# Supplementary material for: Repurposing of the small-molecule adrenoreceptor-inhibitor carvedilol for treatment of the fibrotic lung
Source: Front Pharmacol. 2025 May 22;16:1534989. doi: 10.3389/fphar.2025.1534989 (PMC12137325; doi:10.3389/fphar.2025.1534989)
Supplement: Supplementary file 2 [file DataSheet1.docx]

Supplementary Material

**
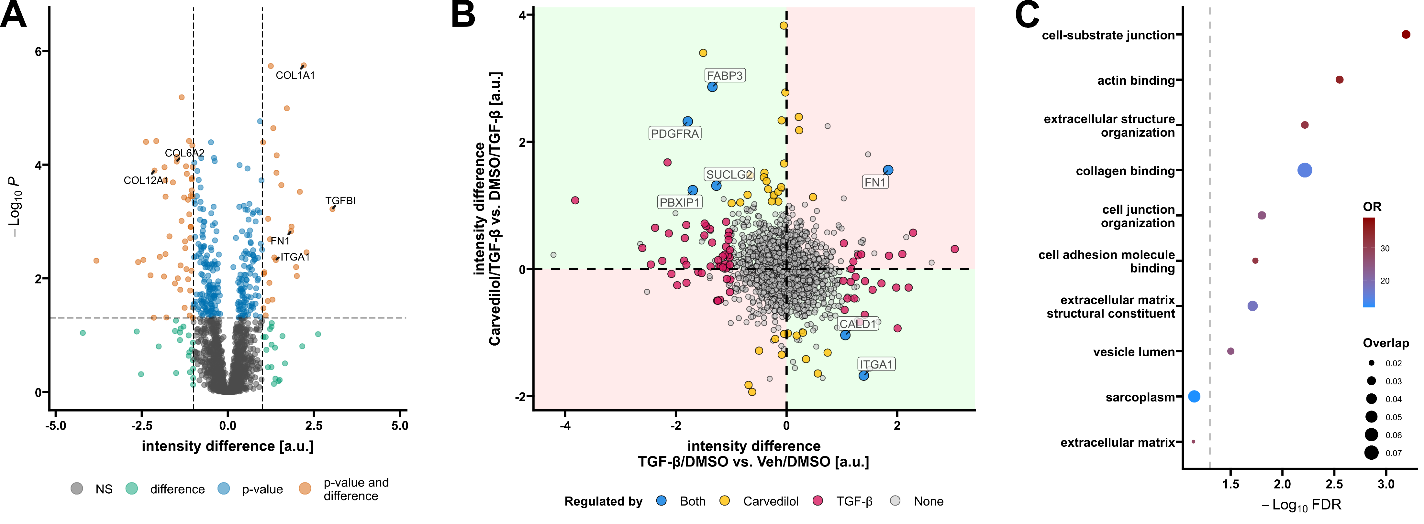
**

**Supplemental Figure 1:** (A) Volcano plots highlighting differential expression of proteins comparing transforming growth factor β (TGFβ) with vehicle (Veh) group (both without carvedilol treatment). Annotated candidates are related to extracellular matrix remodeling and other fibrosis-associated aspects. NS, not significant. (B) Comparison of intensity differences in proteomics data induced by TGFβ (x-axis) and carvedilol (y-axis). Green and red shaded areas in graph highlight adverse and parallel regulated proteins, respectively. (C) Analysis of significantly regulated proteins by TGFβ in MRC-5 for overrepresented gene ontology terms. FDR, false discovery rate; OR, odds ratio; a.u., arbitrary units.
